# Assessment of causal association between differentiated thyroid cancer and disordered serum lipid profile: a Mendelian randomization study

**DOI:** 10.3389/fendo.2023.1291445

**Published:** 2023-12-20

**Authors:** Qiang Ma, Yu Li, Lijuan An, Liang Guo, Xiaokang Liu

**Affiliations:** Department of General Surgery, Lanzhou University Second Hospital, Lanzhou, Gansu, China

**Keywords:** differentiated thyroid cancer, Mendelian randomization, single-nucleotide polymorphisms, exposure factors, disordered serum lipid profile

## Abstract

**Background:**

Research has shown that the disordered serum lipid profile may be associated with the risk of differentiated thyroid cancer (DTC). Whether this association reflect causal effect is still unclear. The aim of this study was to evaluate the causality of circulating lipoprotein lipids on DTC.

**Methods:**

Mendelian randomization (MR) analysis was conducted to evaluate the relationship between the circulating lipoprotein lipids and DTC risk using single-nucleotide polymorphisms (SNPs) from a genome-wide association (GWA) study containing a high-incidence Italian population of 690 cases samples with DTC and 497 controls.

**Results:**

Univariate and multivariate mendelian randomization analysis demonstrated that ‘total cholesterol’, ‘HDL cholesterol’, ‘apolipoprotein B’ and ‘ratio of apolipoprotein B to apolipoprotein A1’ were correlated with DTC. According to sensitivity analysis, our results were reliable. Furthermore, multivariate analysis revealed that there is no causative association between DTC and any of the many cause factors when they interact with one another, suggesting that there was a deep interaction between the four factors, which could affect each other. Finally, the mechanism of the related effects each other as well as the target genes with significant SNP regulatory effects in DTC was explored by conducting functional enrichment analysis and constructing the regulatory networks.

**Conclusions:**

We obtained four exposure factors (total cholesterol, HDL cholesterol, apolipoprotein B and ratio of apolipoprotein B to apolipoprotein A1) closely related to DTC, which laid a theoretical foundation for the treatment of DTC.

## 1 Introduction

The thyroid gland is responsible for the production and release of thyroid hormones and plays an important role in the metabolism, growth, and development of the body (1). The most prevalent endocrine malignancy and the one with the highest rate of growth is thyroid cancer (2). It is more common in women (3), with a 3:1 female-to-male ratio in most geographic regions and demographic groups (4), and it is the fifth most common cancer in women (5). Thyroid cancer affects a younger population than most malignancies. The established risk factors for thyroid cancer include exposure to ionizing radiation, family history, sex, obesity, and alcohol and tobacco use, and relatively little is known about the modifiable risk factors of this disease.

Researchers have found that a disordered serum lipid profile may be related to the thyroid gland (6, 7). There are generally seven serum lipid tests in medicine: triglyceride (TG), total cholesterol (TC), high-density lipoprotein cholesterol (HDL-C), low-density lipoprotein cholesterol (LDL-C), apolipoprotein A1 (Apo A1), apolipoprotein B (Apo B), and ratio of Apo B to Apo A1. A study (8) by Peking Union Medical College Hospital in 2019 showed that most patients with thyroid cancer had dyslipidemia and abnormal indexes: patients with thyroid cancer had higher overall serum lipid levels as well as higher levels of either low-density or high-density cholesterol. It was also found that the levels of Apo A1 and HDL-C in women with thyroid cancer was decreased significantly. According to a study (9) from 2022, thyroid cancer risk is increased by HDL-C, which frequently has low test results. Another research has demonstrated that the risk of thyroid cancer is 39% higher in patients with metabolic syndrome than in the general population. This condition includes abdominal obesity, hypertriglyceridemia, decreased HDL-C, increased blood pressure, and hyperglycemia (10, 11), where patients with TC were divided into three subgroups according to tumor histological analysis: 15 patients with papillary thyroid cancer (PTC), 10 patients with medullary thyroid cancer (MTC), and six patients with follicular thyroid cancer (PTC) were involved in the study. Considering the PTC are commonly grouped together as differentiated thyroid cancer (DTC) (12), the relationship of the progression of DTC and the serum lipid levels has attracted much attention.

Mendelian randomization (MR) (13), which is a method for exploring causality, offering a promising method of examining the effects of modifiable exposures on disease risk, uses a genetic variant as the instrumental variable (IV) in epidemiological studies to mimic a randomized controlled trial (14). In recent years, MR has been favored by the medical research field because of its special advantages and the rapid development of genomics, with the identification of numerous genetic variations closely linked to particular traits in the field of biology and the publication of hundreds of thousands of summary data on the association between exposure, disease, and hereditary variation by genome-wide association studies (GWAS) (15). Although a lot of evidence show that the disordered serum lipid profile is closely related to the risk of thyroid cancer, no study has been able to thoroughly explore the causal relationship between the circulating lipoprotein lipids and thyroid cancer. Therefore, it is necessary to conduct a Mendelian randomized study with a large sample size to explore the risk of multiple circulating lipoprotein lipids and thyroid cancer and to clarify their causal relationship.

This study aimed to obtain the key exposure factors closely related to DTC based on GWAS data on DTC from a previous study (16) (which was conducted in a high-incidence Italian population of 690 cases and 497 controls) through Mendelian randomization analysis, explored the biological processes that they may participate in, and provided some reference for the clinical treatment of thyroid cancer patients.

## 2 Materials and methods

### 2.1 Study design

**Figure 1:**
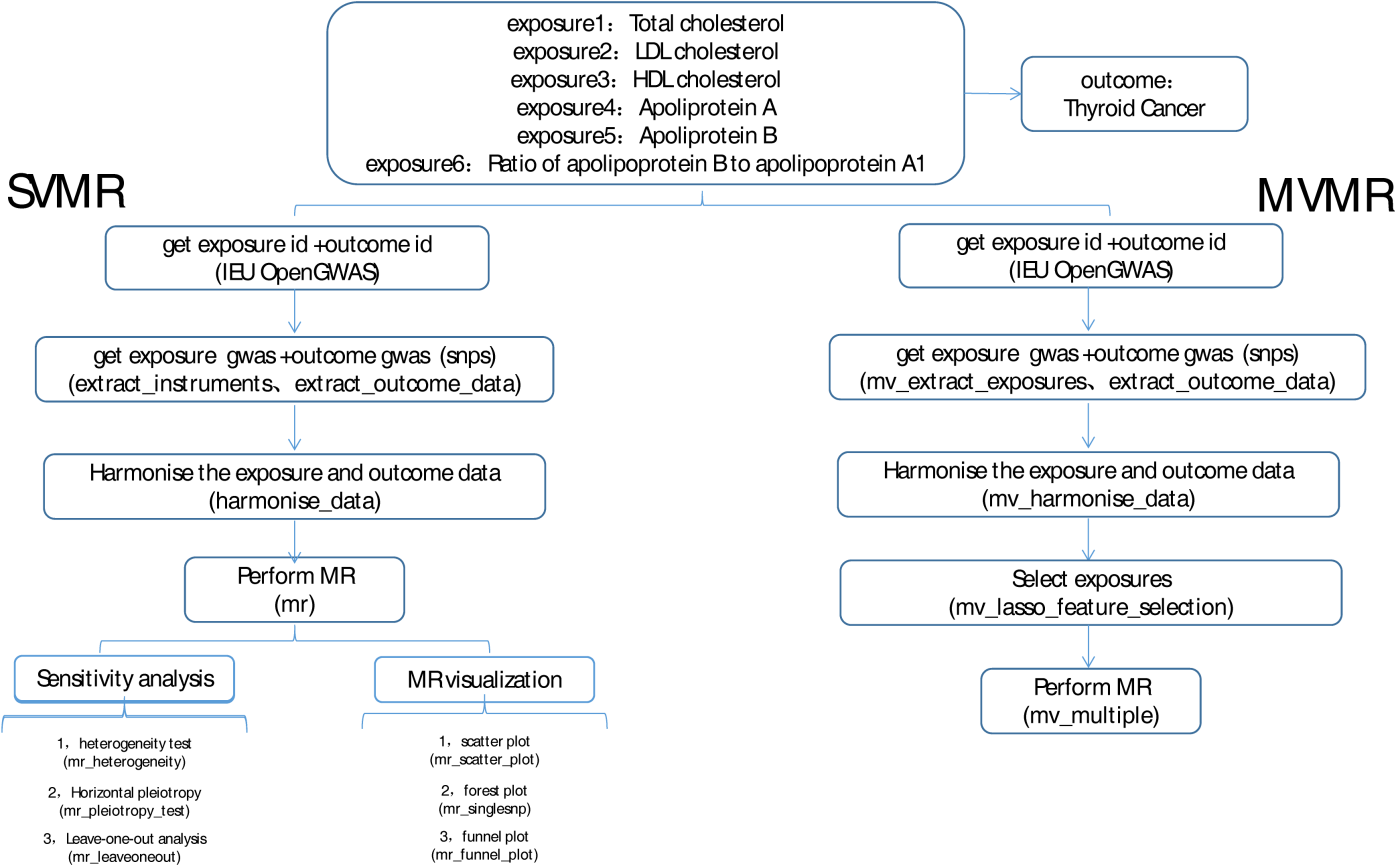
Flow chart of this study.

The overview of the study design was shown in **Figure 1**. MR studies must satisfy the following three assumptions: (a) there is a strong correlation between IVs and exposure, (b) IVs are independent of confounding factors that are related to exposure and outcome, and (c) IVs affect the outcome only through exposure and not through other biological pathways. In our study, we performed MR analyses to analyze the causal relationship between multiple circulating lipoprotein lipids and DTC based on a previous study (16). The study used data from published public databases, and therefore this study did not require any additional ethical approval.

### 2.2 Data acquisition and processing

The GWAS data of exposure factor, including total cholesterol (gwasID: met-d-Total_C; 115,078 samples, 12,321,875 single-nucleotide polymorphisms (SNPs)), LDL cholesterol (gwasID: ebi-a-GCST002222; 94,595 samples, 2,409,690 SNPs), HDL cholesterol (gwasID: ieu-b-4843; 37,120 samples), apolipoprotein A (gwasID: ukb-d-30630_raw; 13,585,234 SNPs), apolipoprotein B (gwasID: ukb-d-30640_raw; 13,585,958 SNPs), ratio of apolipoprotein B to apolipoprotein A1 (gwasID: met-d-ApoB_by_ApoA1; 115,078 samples, 12,321,875 SNPs), and outcomes (where 649 DTC samples, 431 controls, and 572,028 SNPs were involved in the study), were obtained from published GWAS by IEU Open GWAS database (https://gwas.mrcieu.ac.uk/). The function of extract instruments in the “Two Sample MR” package was used to search SNP that were significantly associated with exposure factors. The value of *p <*5 * 10^-8^ was determined as the threshold. Pheno Scanner was used to remove the SNP loci that might have potential confounding effects on outcomes. The clump = TRUE was selected to remove the instrumental variable (SNP) with linkage disequilibrium (LD) (*r* ^2 = ^0.001), kb = 10,000.

### 2.3 Univariate and multivariate Mendelian randomization analysis

After the IVs were filtered, MR analyses were performed by combining the MR function and five methods as follows: MR Egger, weighted median, inverse variance weighted (IVW), simple mode, and weighted mode. The results were mainly referenced to IVW. Then, odds ratios (ORs) were calculated, with values greater than 1 being a risk factor and those less than 1 being a protective factor. The results were presented using scatter plots, forest plots, and funnel plots.

To determine the reliability of the results of the analysis, a sensitivity analysis was conducted via a heterogeneity test, the horizontal pleiotropy test, and the leave-one-out (LOO) method. When performing the heterogeneity test, a *Q*-value greater than 0.05 indicates that there is no heterogeneity. A *P*-value that is greater than 0.05 demonstrates that there is no horizontal pleiotropy in the horizontal pleiotropy test. The LOO is used to see if there are outliers in the effect of each SNP. Then, all the factors considered to be correlated were included in the multivariate MR analysis.

### 2.4 Functionality analysis of IVs of exposure factors with causal effect on thyroid cancer

In order to investigate the functionality of the IVs of exposure factors related to thyroid cancer, SNP data obtained by MR analysis was integrated with the cis-expression quantitative trait loci (cis-eQTL) data in the eQTLGen (https://eqtlgen.org/) database, and the target genes with significant SNP regulatory effects were screened for functional annotation analysis.

For the potential function and singling pathway, “clusterProfiler” R package was used to perform Gene Ontology (GO) and Kyoto Encyclopedia of Genes and Genomes (KEGG) enrichment analyses on the target genes. Meanwhile, the protein–protein interaction (PPI) information among the target genes was obtained from the STRING database (https://string-db.org/) with a medium confidence level greater than 0.4, and the interaction network was imported into the Cytoscape software for visualization (removing isolated targets), where the core co-expression network was further screened by the MCODE plug-in to obtain key genes. Furthermore, the transcription factors (TFs) corresponding to the key genes were predicted using the JASPER database in the NetworkAnalyst (https://www.networkanalyst.ca/) for the investigation of the regulatory mechanism.

## 3 Results

### 3.1 Selection of genetic instruments

**Figure 2:**
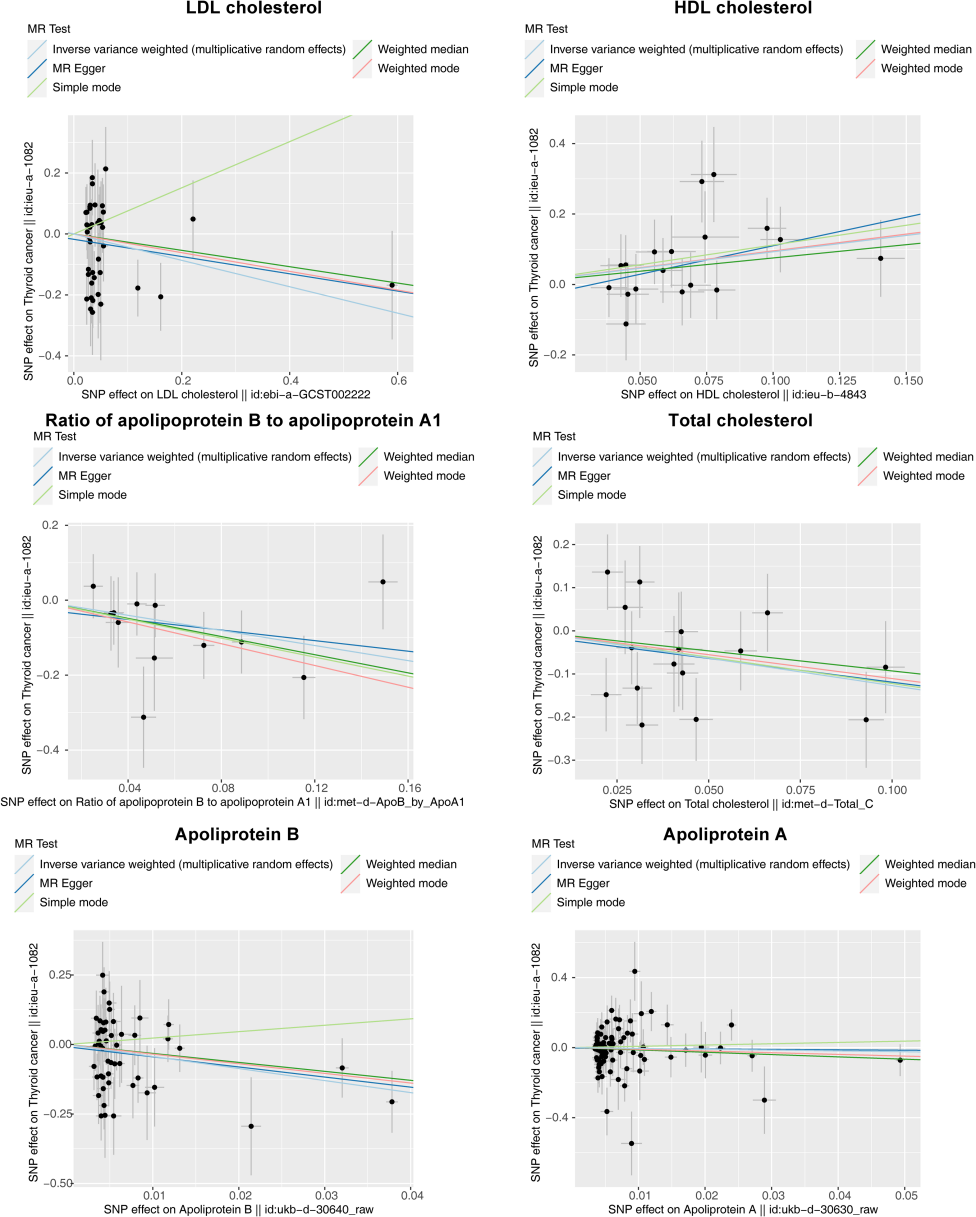
Scatter plots of Mendelian randomization (MR) analysis for the relationship of six exposure factors (total cholesterol, LDL cholesterol, HDL cholesterol, apolipoprotein A, apolipoprotein B, and ratio of apolipoprotein B to apolipoprotein A1) and outcomes (thyroid cancer). The X-axes show the SNP-exposure effect and the Y-axes show the SNP-outcome effect. The inverse variance weighted method is mainly concerned. The small intercept indicates that the analysis is less affected by confounding factors to ensure reliability. The slope of each line shows the estimated causal effect of cholesterol-related factor on thyroid cancer, where a positive slope reflects a positive link with the risk of thyroid cancer (risk factor), and a negative slope reflects a reverse causality (protective factor).

**Figure 3:**
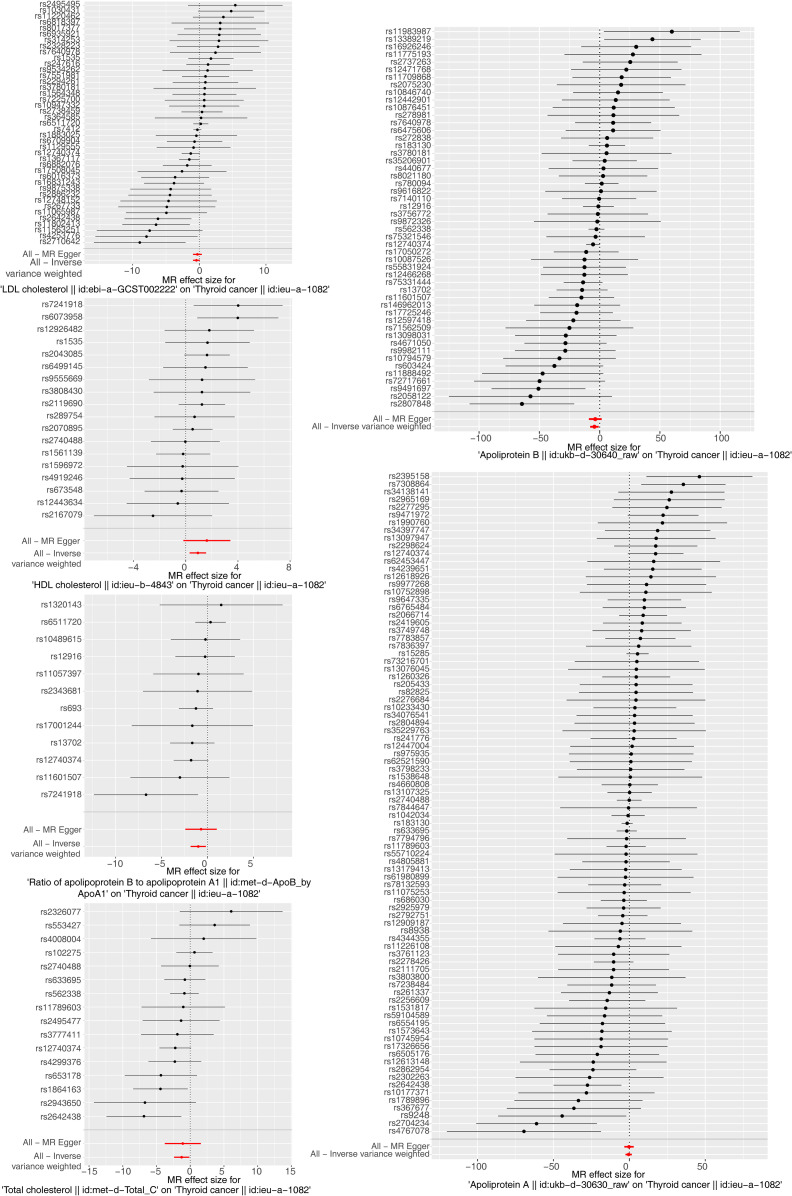
Forest plots of Mendelian randomization analysis for the relationship of six exposure factors and thyroid cancer combining a Wald ratio method for each SNP effect (horizontal black solid line) and inverse variance weighted for fixed-effects (horizontal red solid line). The solid line completely on the left side of 0 indicates that this SNP estimates that exposure factors can reduce the risk of disease, the solid line completely on the right side of 0 indicates that this SNP estimates that exposure factors can increase the risk of disease, and the results of those crossing 0 are not significant.

**Figure 4:**
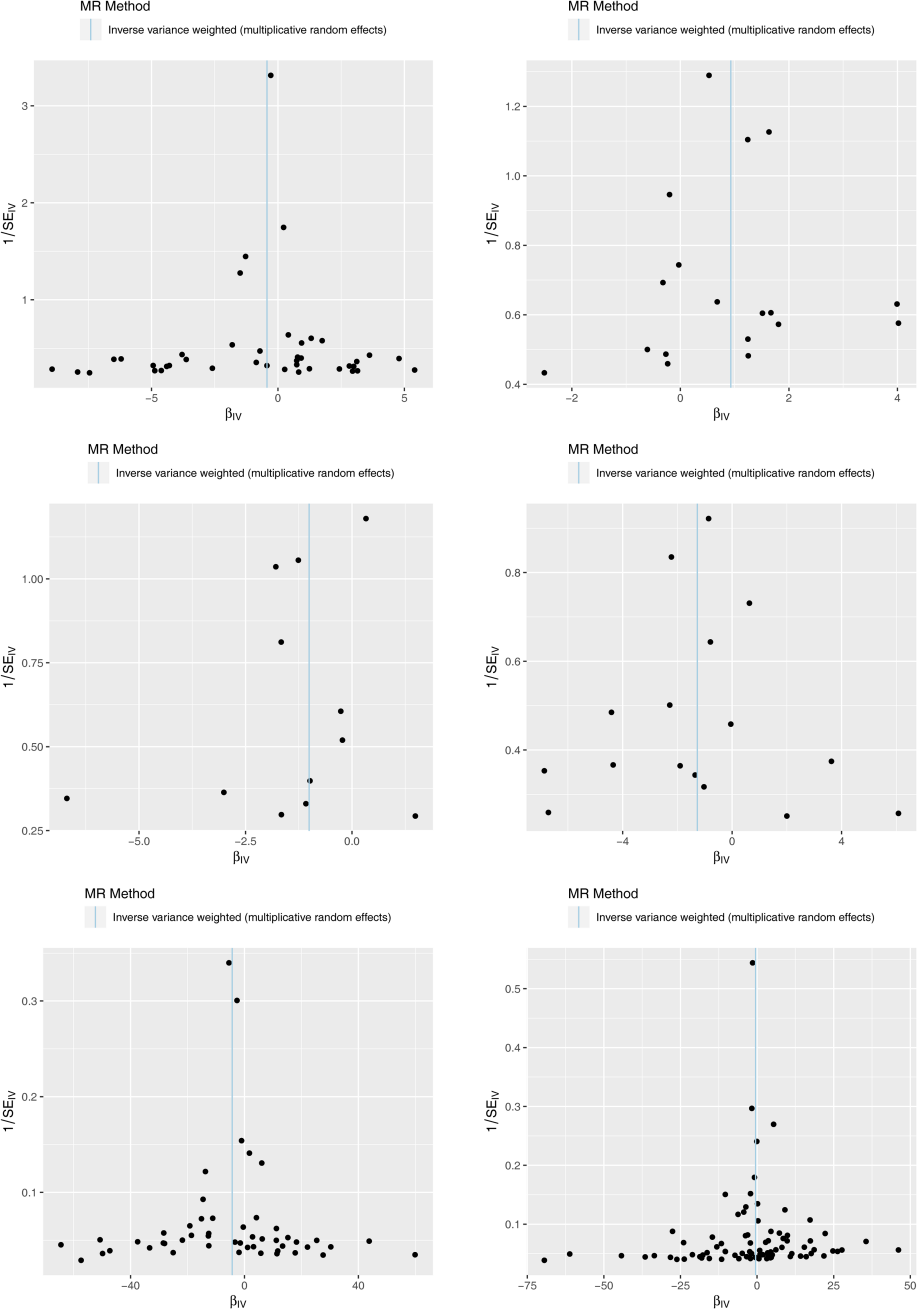
Funnel plots of Mendelian randomization (MR) analysis for six exposure factors. The SNPs are symmetrically distributed along both sides of the inverse variance weighted line, indicating MR links to Mendel’s second law.

**Figure 5:**
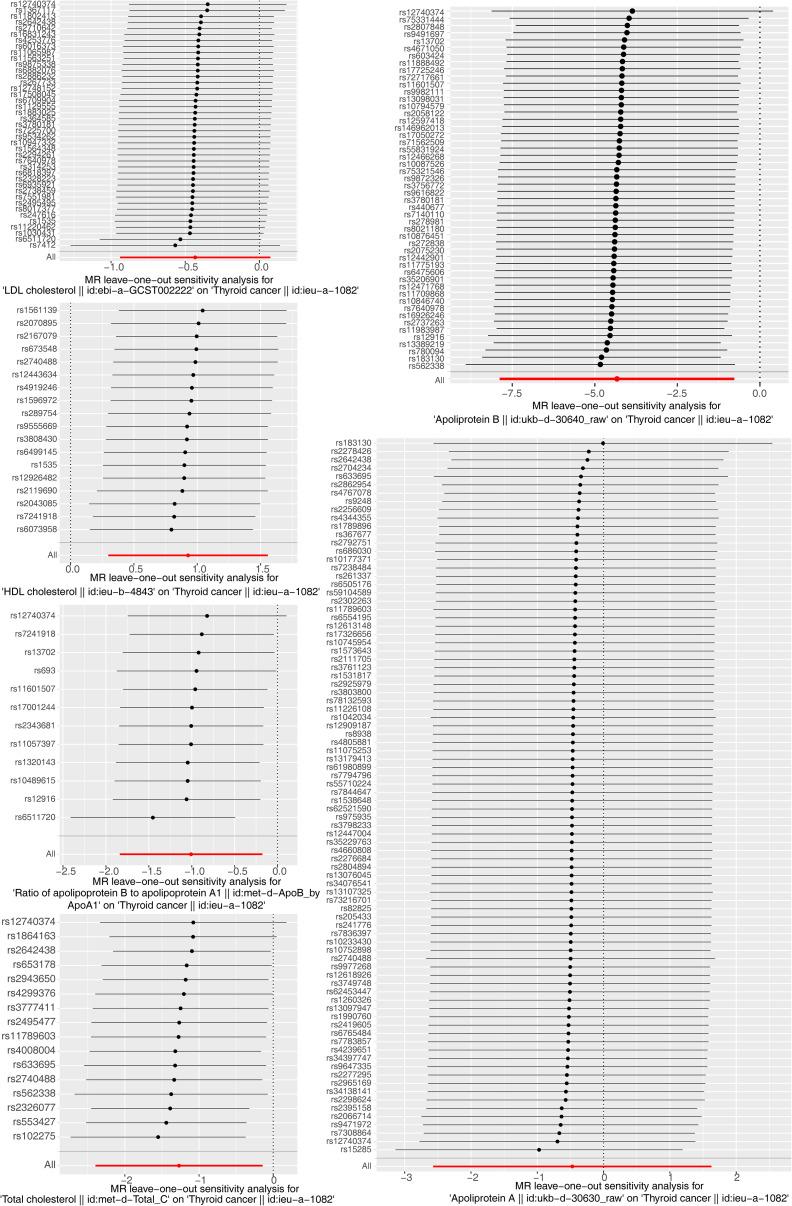
Forest plot of the leave-one-out (LOO) test for sensitivity tests. Calculate the Mendelian randomization (MR) results of the remaining SNPs after removing the SNPs one by one. The smooth black dot line reflects the robustness of the MR results.

The results of the MR analysis corresponding to all exposure factors are shown in **Supplementary Table S1** where the results of IVW were mainly used to explain the findings, supplemented by four other methods. It visualized the effect measure of each exposure factor on outcome and estimated 95% confidence interval (CI) of individual studies, and it revealed that the concentration of “total cholesterol” (*p* = 0.01, OR = 0.281, 95% CI: 0.107–0.736), “HDL cholesterol” (*p* = 0.004, OR = 2.530, 95% CI: 1.347–4.750), “apolipoprotein B” (*p* = 0.007, OR = 0.013, 95% CI: 0.001–0.299), and “ratio of apolipoprotein B to apolipoprotein A1” (*p* = 0.017, OR = 0.365, 95% CI: 0.159–0.838), respectively, was causally correlated with thyroid cancer. Observing the identified OR values, HDL cholesterol might be the risk factor (OR > 1, *p* < 0.05) and “total cholesterol”, “apolipoprotein B”, and “ratio of apolipoprotein B to apolipoprotein A1” were the protective factors (OR < 1, *p* < 0.05) for thyroid cancer. The scatter plot indicated that the intercept of the IVW line was very small, indicating the exclusion of confounding factors and the robustness of the analysis results. Meanwhile, the slope of the line for “total cholesterol”, “apolipoprotein B”, and “ratio of apolipoprotein B to apolipoprotein A1”, respectively, was negative, suggesting that an increase of those apolipoprotein led to a reduced risk of thyroid cancer. However, HDL cholesterol showed the opposite trend, which echoed the findings shown in **Figure 2**. Furthermore, the location of the concentration points of the different correlation factors was shown in the forest map drawn by SNP to explore the diagnostic efficacy of each exposure factor on outcome. By comprehensively observing the estimated causal effect of individual SNP (black) and overall effect of exposure (red), the risk relationship between each exposure factor and outcome supported a previous point of view (**Figure 3**). The funnel plot showed that MR conformed to Mendel’s second law of random grouping (**Figure 4**). Additionally, the Cochran’s *Q* test was used to test the heterogeneity of exposure with *p* < 0.05, where LDL cholesterol was detected with IVW *p* = 0.016; hence, the random-effects model was utilized. Meanwhile, the fixed-effects model was utilized for other exposures in which no substantial heterogeneity (*p* > 0.05) was observed. Furthermore, there was no horizontal pleiotropy in all IVs (*p* > 0.05) (**Supplementary Tables S2**, **S3**). The forest plot of the LOO method suggested that all error lines were to the left of 0, indicating that there were no points of deviation (**Figure 5**). In summary, there was a causal relationship between serum lipid profile and thyroid cancer.

### 3.2 Key exposure factors for multivariate analysis

**Figure 6:**
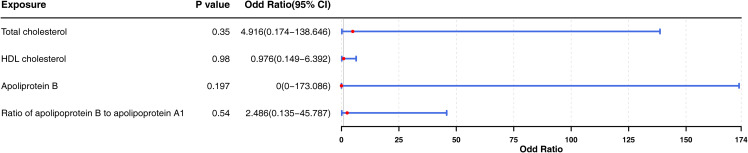
Forest plot for multivariable Mendelian randomization analyses by adjusting four exposure factors associated to the probability of thyroid cancer (“total cholesterol”, “HDL cholesterol”, “apolipoprotein B”, and “ratio of apolipoprotein B to apolipoprotein A1”) together for the risk of thyroid cancer. OR, odds ratio; CI, confidence intervals. According to the *P*-values of four factors, which had no significance.

Subsequently, we included “total cholesterol”, “HDL cholesterol”, “apolipoprotein B”, and “ratio of apolipoprotein B to apolipoprotein A1” in the multivariate MR analysis (**Figure 6**). There is no causal relationship between the four factors and thyroid cancer (*p* > 0.05), suggesting a potential interaction among these factors and that they could influence each other.

### 3.3 Investigation of the molecular mechanisms of four exposure factors for thyroid cancer

After searching the target genes with significant SNP regulatory effects which were integrated with cis-eQTL data in the eQTLGen database (see **Supplementary Tables S4**-**S7**), the molecular mechanisms of the IVs of four exposure factors (ieu-b-4843: HDL cholesterol; met-d-ApoB_by_ApoA1: ratio of apolipoprotein B to apolipoprotein A1; met-d-Total_C: total cholesterol; and ukb-d-30640_raw: apolipoprotein B) on thyroid cancer were explored by conducting a functional enrichment analysis and constructing a PPI network and a TF–gene network.

**Figure 7:**
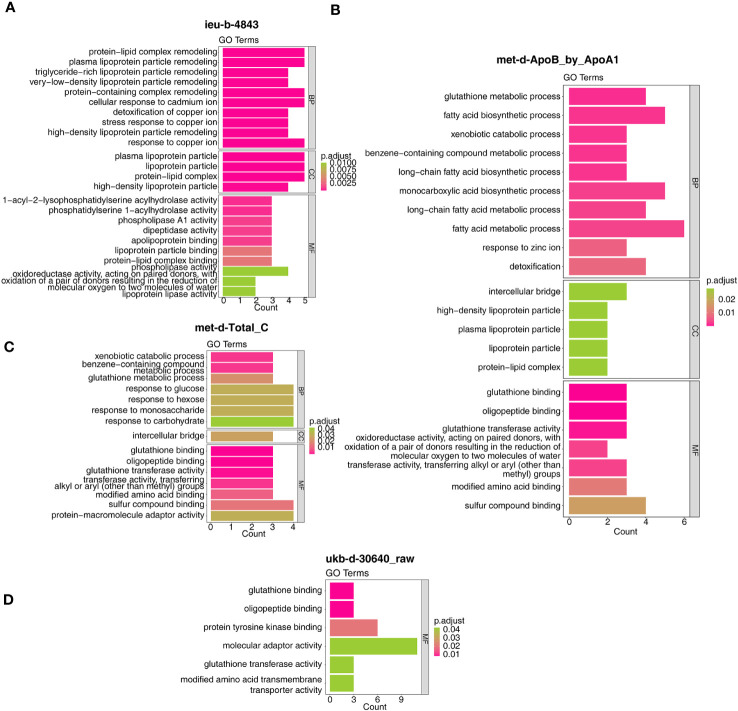
Gene Ontology analysis of the target genes. **(A)** ieu-b-4843: HDL cholesterol. **(B)** met-d-ApoB_by_ApoA1: ratio of apolipoprotein B to apolipoprotein A1. **(C)** met-d-Total_C: total cholesterol. **(D)** ukb-d-30640_raw: apolipoprotein B.

**Figure 8:**
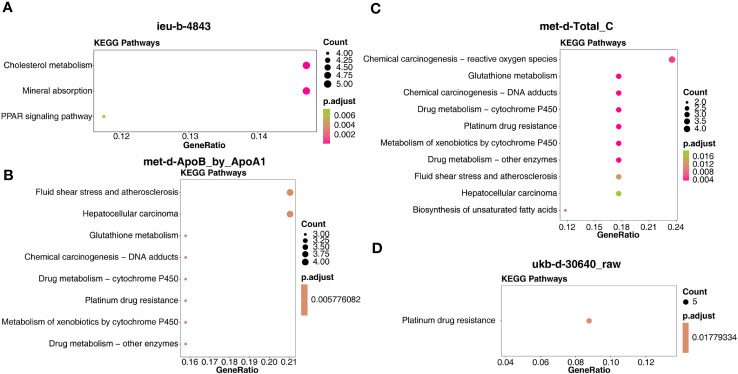
Kyoto Encyclopedia of Genes and Genomes enrichment analysis of the target genes. **(A)** ieu-b-4843: HDL cholesterol. **(B)** met-d-ApoB_by_ApoA1: ratio of apolipoprotein B to apolipoprotein A1. **(C)** met-d-Total_C: total cholesterol. **(D)** ukb-d-30640_raw: apolipoprotein B.

In detail, the target genes of ieu-b-4843 were principally associated with the terms of lipoprotein particle remodeling and response to copper ion. The target genes of the three other IVs were common to the GO-molecular functions (MF) of glutathione binding, glutathione transferase activity, and oligopeptide binding (**Figures 7A–D**). Meanwhile, the target genes regulated by ieu-b-4843 mainly enriched the KEGG pathways of cholesterol metabolism, mineral absorption, and PPAR signaling pathway (**Figure 8A**), and the target genes of met-d-ApoB_by_ApoA1, met-d-Total_C, and ukb-d-30640_raw might be commonly involved in platinum drug resistance (**Figures 8B–D**).

**Figure 9:**
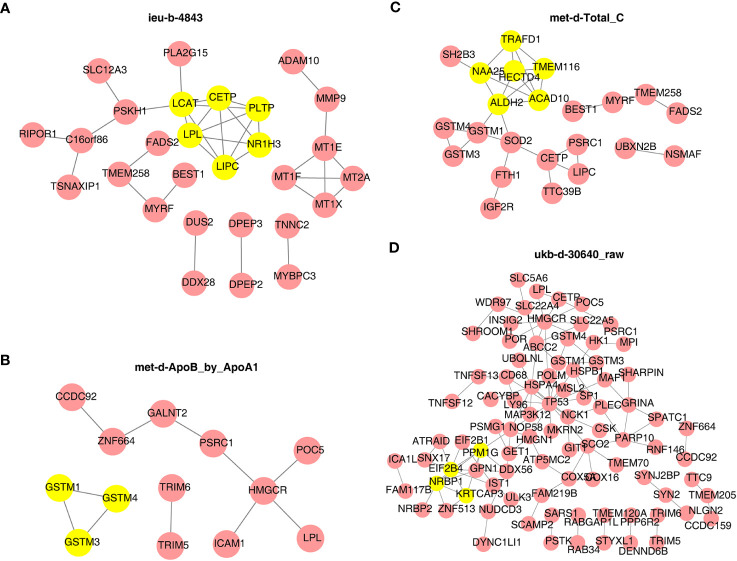
Protein–protein interaction network of target genes and identity of core key genes. **(A)** ieu-b-4843: HDL cholesterol. **(B)** met-d-ApoB_by_ApoA1: ratio of apolipoprotein B to apolipoprotein A1. **(C)** met-d-Total_C: total cholesterol. **(D)** ukb-d-30640_raw: apolipoprotein B.

Next, the PPI network in **Figure 9** indicated that LCAT, CETP, PLTP, LPL, LIPC, and NR1H3 were the core key genes for ieu-b-4843 and interacted with each other (**Figure 9A**). GSTM1, GSTM4, and GSTM3 were closely related to met-d-ApoB_by_ApoA1 (**Figure 9B**). HECTD4, TRAFD1, ACAD10, NAA25, TMEM116, and ALDH2 were likewise regulated by met-d-Total_C (**Figure 9C**). PPM1G, EIF2B4, NRBP1, and KRTCAP3 were considered as the core genes for ukb-d-30640_raw (**Figure 9D**).

**Figure 10:**
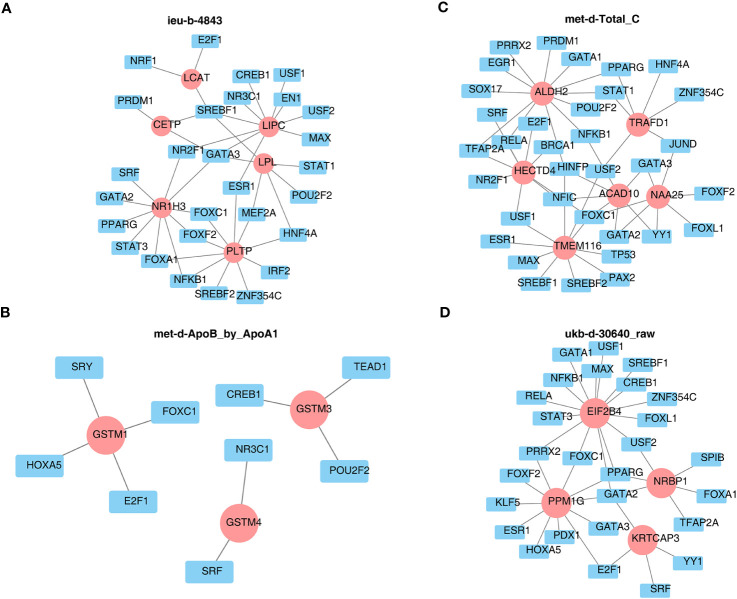
Prediction of the transcription factor networks targeting key genes. **(A)** ieu-b-4843: HDL cholesterol. **(B)** met-d-ApoB_by_ApoA1: ratio of apolipoprotein B to apolipoprotein A1. **(C)** met-d-Total_C: total cholesterol. **(D)** ukb-d-30640_raw: apolipoprotein B.

Furthermore, the TF site targeting these key genes were predicted and are shown in **Figure 10**, such as SREBF1, GATA3 for more than two key genes of ieu-b-4843 (**Figure 10A**), USF2, RELA, HINFP, FOXC1 for various key genes of met-d-Total_C (**Figure 10C**), and GATA2 for all key genes of ukb-d-30640_raw (**Figure 10D**). Meanwhile, the TFs of each key gene of met-d-ApoB_by_ApoA1 are exhibited in **Figure 10B**.

## 4 Discussion

In China, more than 40% of adults suffer from dyslipidemia. The normal level of serum lipid can maintain the normal physiological function of the body (17). Once the serum lipid level is abnormal, it may cause cardio-cerebral vascular disease, fatty liver, infection, trauma, etc. (18). With the continuous study of dyslipidemia, studies at home and abroad have reported that dyslipidemia can increase the risk of malignant tumors. Dyslipidemia was an important part of metabolic syndrome. In this context, the study of the correlation between serum lipid levels and thyroid cancer has attracted extensive attention. The formation and occurrence of malignant tumors have been linked to serum lipid levels, according to years of research by specialists and academics from different nations. As part of the metabolic syndrome, several clinical and molecular pathways suggested a connection between triglycerides and cancer risk (19). Research showed that the increase of serum TC level leads to an increased risk of rectal cancer, colon cancer, prostate cancer, and testicular cancer. There was a positive correlation between serum triglycerides and esophageal, colon, rectum, lung, kidney, and thyroid cancer (20). Existing research found that triglycerides were a positive risk factor for male thyroid cancer (21). Studies have shown that men with high levels of triglycerides have a higher risk of developing thyroid cancer than women.

Some studies (8, 22, 23) have studied the relationship between TC and thyroid cancer risk. Compared with the control group, the levels of TC in thyroid cancer cases were similar or higher. On the other hand, in some previous studies, the lower levels of TC in thyroid cancer cases might be partially attributed to a reverse causal relationship, where thyroid cancer patients showed a decrease in all lipid biomarker (including TC) levels studied in the decade before cancer diagnosis, while the control group showed a sustained increase in TC levels during the same period due to aging. A large-scale cohort study based on the Swedish AMORIS cohort with 30 years of follow-up found that a higher level of TC and HDL-C was negatively correlated with a lower risk of thyroid cancer (7). However, a recent meta-analysis of 42 studies showed that there was no relationship between TC risk and dyslipidemia (10).

According to our research, greater levels of TC were favorably connected with the development of thyroid cancer, while higher levels of HDL-C were adversely correlated with the development of thyroid cancer. We could simply speculate that HDL-C was a kind of cholesterol carried by high-density lipoproteins, which transfer between the peripheral blood and liver. HDL-C was known to prevent cardiovascular diseases (24), and lower levels of HDL-C were also considered to be associated with higher risks of blood malignancies, nervous system cancers, and breast cancer (25). Given that HDL-C was a key regulator of innate and adaptive immune responses and has antioxidant, antiapoptotic, and anti-inflammatory properties, it might indeed have a preventive effect on cancer. This was suggested by a cross-sectional or case–control study (26, 27) that found that thyroid cancer cases had lower HDL-C levels than the control group.

In addition to their general carcinogenic effects, TC and HDL-C might also have a preventive effect on thyroid cancer, especially due to the activity of the thyroid and the hormones. An important function of the thyroid gland was to regulate cholesterol metabolism. Higher levels of cholesterol might indicate hypothyroidism (28), while lower levels of cholesterol might indicate hyperthyroidism—for example, it has been proposed that up to 13% of patients with hyperlipidemia suffer from hypothyroidism (29, 30). Given the findings of this study, individuals with lower levels of long-term TC and HDL-C might exhibit a more active thyroid function, which might, in turn, activate a series of cellular signaling pathways and lead to carcinogenesis. Finally, although the incidence rate of thyroid cancer was different between men and women, the association between TC, HDL-C, and thyroid cancer seems to be similar between men and women in the current study.

Our study also found that a high level of Apo B and Apo B/Apo A1 ratio was positively related to the occurrence of thyroid cancer. Given that there were relatively few studies on Apo A, Apo B, and Apo B/Apo A1 ratio (8, 31), more studies need to verify the ineffective results of these thyroid cancer biomarkers in this study. Our research results required further extensive research to verify.

We also performed a multi-factor MR analysis, and the results revealed that there is no causative association between thyroid cancer and any of the many cause factors when they interact with one another. In a multivariate MR analysis, it is important to note that different exposure factors can interact with each other, particularly if there is a biological association between them. Consequently, this interaction mechanism can result in insignificant effects because a multivariate analysis aims to consider the simultaneous relationship among multiple factors, thereby diminishing the independent impact of each individual factor on outcome. This suggested that TC, HDL-C, Apo B, and Apo B/Apo A1 ratio had a deep interaction relationship with each other and could influence each other.

Next, the mechanism that has related effects on each other as well as the target genes, with significant SNP regulatory effects in thyroid cancer, was explored by conducting a functional enrichment analysis and constructing a PPI network and a TF–gene network. Beata Wojtczak et al. (32) has found that metallothioneins (MTs) are involved in many cellular processes, such as the binding and transport of zinc and copper ions, and the expression of functional MT genes may contribute to thyroid carcinogenesis. Jorge Gaspar et al. (33) believes that combining glutathione S-transferase (GST) polymorphisms can lead to a moderate increase in the risk of thyroid cancer, especially in the papillary type. GSTP1 polymorphisms may regulate the onset age of the disease. The functional enrichment results here correspond exactly to the screening results of the PPI module genes later [met d ApoB by ApoA1: all three module genes (GSTM1, GSTM4, and GSTM3) are transcripts encoding Mu-type GST]. Elias Mazokopakis (34) believes that the prescription of supplements with organic forms of Se must be preferred over supplements with inorganic forms of Se among DTC patients for the protection of their salivary glands from ^131^I treatment, and Brazil nuts could be another choice. Eman A. Toraih et al. (35) has evaluated the prognostic value of PPARα/RXRα tissue expression in patients with thyroid carcinoma. Some studies found that LPL, FATP2, and CPT1A can all promote the migration of thyroid cancer cells (36), while USF-2 inhibits the proliferation of normal thyroid cells and thyroid cancer cells (37). RELA and TP53 are involved in the anti-thyroid cancer mechanism of triptolide (38). In addition, there is a research (39) which has found that HINFP, FOXC1, and GATA2, as transcription factors, may be involved in different molecules between FTC and benign follicular thyroid adenoma. However, a large amount of research is still needed to conduct deeper research on the relevant mechanisms.

According to our findings, dyslipidemia can both trigger and accelerate the growth of thyroid cancer, and aberrant thyroid function, particularly decreased, can also encourage a rise in the production of liver cholesterol. Therefore, thyroid issues could be checked first if dyslipidemia or metabolic abnormalities were discovered during a physical examination. If there were nodules in the thyroid gland at this time, it indicated that metabolism was affected to a certain extent, and the next decision needed to be made based on the situation. For people with thyroid nodules, when it was difficult to evaluate and differentiate between benign and malignant nodules, they can expand at the same time, detect seven serum lipids, and observe the metabolism of patients. This nodule will likely be thyroid cancer and have some effect on the body’s metabolism if the ultrasonic features suggest malignancy, TC rises, and HDL-C continues to fall.

Our MR study used genetic data as a bridge to explore the causal relationship between serum lipids and thyroid cancer. This study was based on one or more alleles that affect risk factors and involved the “randomization” of participating genes to determine whether carriers of these genetic variations have different disease risk. Moreover, we have demonstrated through various methods that our research results were not affected by confounding factors and conducted heterogeneity tests. Thus, these findings have significant clinical significance. Firstly, these findings provide potential biomarkers that can be directly detected in clinical practice for the prevention and diagnosis of thyroid cancer. By monitoring and regulating these exposure factors, doctors can detect and intervene in the development of thyroid cancer. Secondly, understanding the impact of these exposure factors on the development of thyroid cancer and adjusting the treatment based on the specific exposure factor levels of the patients may improve the treatment effectiveness, thereby helping doctors develop more personalized and effective treatment plans. In addition, by understanding the relationship between these exposure factors and thyroid cancer, the public can adopt a positive lifestyle and behavior, maintain a healthy diet, engage in appropriate exercise, and undergo regular examinations to reduce the risk of illness. In summary, the discovery of four exposure factors closely related to thyroid cancer by Mendelian randomization analysis has a significant clinical significance and has a positive impact on the prevention, diagnosis, and treatment of thyroid cancer as well as in raising public health awareness. On the other hand, our research also has limitations, as MR may have errors and MR statistics also have limitations and as the given genetic variations typically only explain a small portion of the risk factors. In addition, we could verify the association between HDL-C and TC with thyroid cancer through current research, but we cannot verify the impact of other biomarkers (such as Apo B and Apo B/Apo A1 ratio) on the studied association. Therefore, detailed data research on these factors is needed in the future to better understand the potential mechanisms of the current research results.

In conclusion, based on the public GWAS data on DTC, our analysis demonstrated that TC, HDL-C, ApoB, and the ratio of ApoB to ApoA1 are four exposure factors closely related to DTC, in which HDL-C might be the risk factor and TC, ApoB, and the ratio of ApoB to ApoA1 were the protective factors. There was no heterogeneity and horizontal pleiotropy of each exposure, while it is suggested that TC, HDL-C, Apo B, and Apo B/Apo A1 ratio had a deep interaction relationship with each other and could influence each other using a multi-factor MR analysis. Moreover, combining the target genes with significant SNP regulatory effects in the eQTLGen database, the molecular mechanisms of the IVs of four exposure factors on thyroid cancer were further explored with the functional annotation and the regulatory networks, providing additional insights into the study of thyroid cancer.

## Data availability statement

The datasets presented in this study can be found in online repositories. The names of the repository/repositories and accession number(s) can be found in the article/**Supplementary Material**.

## Author contributions

QM: Conceptualization, Data curation, Formal Analysis, Funding acquisition, Writing – original draft. YL: Resources, Software, Writing – original draft. LA: Data curation, Formal Analysis and editing. LG: Data curation, Funding acquisition, Investigation, Project administration, Writing – review & editing. XL: Methodology, Validation, Writing – review & editing.

## Supplementary material

Supplementary Table 4 Lists of target genes of ieu-b-4843 within cis-expression quantitative trait loci (cis-eQTL) data.

Supplementary Table 5 Lists of target genes of met-d-ApoB_by_ApoA1 within cis-eQTL data.

Supplementary Table 6 Lists of target genes of met-d-Total_C within cis-eQTL data.

Supplementary Table 7 Lists of target genes of ukb-d-30640_raw data.
